# Effects of Antibody Response after Booster Vaccination on SARS-CoV-2 Breakthrough Infections and Disease Outcomes in Advanced Cancer Patients: A Prospective Analysis of the Vax-on-Third Study

**DOI:** 10.3390/curroncol30050386

**Published:** 2023-05-17

**Authors:** Fabrizio Nelli, Agnese Fabbri, Antonella Virtuoso, Diana Giannarelli, Julio Rodrigo Giron Berrios, Eleonora Marrucci, Cristina Fiore, Marta Schirripa, Carlo Signorelli, Mario Giovanni Chilelli, Francesca Primi, Gloria Pessina, Federica Natoni, Maria Assunta Silvestri, Enzo Maria Ruggeri

**Affiliations:** 1 Department of Oncology and Hematology, Medical Oncology Unit, Central Hospital of Belcolle, 01100 Viterbo, Italy; 2 Biostatistics Unit, Scientific Directorate, Fondazione Policlinico Universitario A. Gemelli, IRCCS, 00168 Rome, Italy; 3 Department of Oncology and Hematology, Molecular Biology and Covid Diagnostics, Central Hospital of Belcolle, 01100 Viterbo, Italy; 4 Department of Oncology and Hematology, Microbiology and Virology Unit, Central Hospital of Belcolle, 01100 Viterbo, Italy

**Keywords:** SARS-CoV-2, COVID-19, tozinameran, vaccination, third dose, breakthrough infection, solid tumors, advanced disease, active treatment

## Abstract

(1) Background: The clinical implications of COVID-19 outbreaks following SARS-CoV-2 vaccination in immunocompromised recipients are a worldwide concern. Cancer patients on active treatment remain at an increased risk of developing breakthrough infections because of waning immunity and the emergence of SARS-CoV-2 variants. There is a paucity of data on the effects of COVID-19 outbreaks on long-term survival outcomes in this population. (2) Methods: We enrolled 230 cancer patients who were on active treatment for advanced disease and had received booster dosing of an mRNA-BNT162b2 vaccine as part of the Vax-On-Third trial between September 2021 and October 2021. Four weeks after the third immunization, IgG antibodies against the spike receptor domain of SARS-CoV-2 were tested in all patients. We prospectively evaluated the incidence of breakthrough infections and disease outcomes. The coprimary endpoints were the effects of antibody titers on the development of breakthrough infections and the impact of COVID-19 outbreaks on cancer treatment failure. (3) Results: At a median follow-up of 16.3 months (95% CI 14.5–17.0), 85 (37%) patients developed SARS-CoV-2 infection. Hospitalization was required in 11 patients (12.9%) and only 2 (2.3%) deaths related to COVID-19 outbreaks were observed. Median antibody titers were significantly lower in breakthrough cases than in non-cases (291 BAU/mL (95% CI 210–505) vs. 2798 BAU/mL (95% CI 2323–3613), *p* < 0.001). A serological titer cut-off below 803 BAU/mL was predictive of breakthrough infection. In multivariate testing, antibody titers and cytotoxic chemotherapy were independently associated with an increased risk of outbreaks. Time-to-treatment failure after booster dosing was significantly shorter in patients who contracted SARS-CoV-2 infection (3.1 months (95% CI 2.3–3.6) vs. 16.2 months (95% CI 14.3–17.0), *p* < 0.001) and had an antibody level below the cut-off (3.6 months (95% CI 3.0–4.5) vs. 14.6 months (95% CI 11.9–16.3), *p* < 0.001). A multivariate Cox regression model confirmed that both covariates independently had a worsening effect on time-to-treatment failure. (4) Conclusions: These data support the role of vaccine boosters in preventing the incidence and severity of COVID-19 outbreaks. Enhanced humoral immunity after the third vaccination significantly correlates with protection against breakthrough infections. Strategies aimed at restraining SARS-CoV-2 transmission in advanced cancer patients undergoing active treatment should be prioritized to mitigate the impact on disease outcomes.

## 1. Introduction

Compelling evidence has established the safety and efficacy of SARS-CoV-2 mRNA-based vaccinations in immunocompromised individuals [1]. However, cancer patients diagnosed with advanced disease areat increased risk of developing COVID-19 breakthrough infections and severe outcomes [2,3,4]. In this population, intrinsic immunosuppression and the receipt of antineoplastic agents have been associated with poorer immunologic responses [5,6]. A third dose of an mRNA-BNT162b2 vaccine (tozinameran) has proven to elicit a stronger antibody response than the initial two-dose series in most patients with solid malignancies receiving active treatment [7,8,9,10,11]. A viable recall of T-cell-mediated responses is thought to underlie the enhancement of humoral immunogenicity [12]. Subsequent retrospective studies have confirmed that third booster vaccinations improved the short-term clinical outcomes of cancer patients by reducing 30-day mortality and hospitalizations [13,14]. Population-based studies have found that cancer patients continue to be at an increased risk of COVID-19 outbreaks even after triple vaccination due to waning immunity and the emergence of immune-evading variants of concern (VOCs). The cumulative risk of SARS-CoV-2 infections after booster vaccination was found to be higher in patients with pancreatic, liver, lung, and colorectal cancer. Lower odds were observed in recipients with breast, gynecological, and prostate cancer. These findings suggest that cancer as a disease entity is a risk factor for breakthrough infections in vaccinated patients, with heterogeneous effects depending on specific cancer types and presumably different active treatments [15]. In addition, mild SARS-CoV-2 infections and restraining measures may disrupt the regularity of antineoplastic treatment, leading to a potentially worsening effect on survival outcomes [16]. Limited data are also available about the capabilities of antibody responses induced by booster vaccinations in preventing breakthrough infections over the long term [17,18]. Here, we analyzed the incidence of SARS-CoV-2 infections in a prospective cohort of cancer patients with actively treated advanced disease who had participated in the Vax-On-Third study. We sought to evaluate the effects of humoral responses after the third dose of tozinameran on COVID-19 outbreaks and their impact on cancer treatment failure.

## 2. Materials and Methods

### 2.1. Study Design and Participants

The Vax-On-Third study is a prospective, observational cohort study with a previously reported design and primary results [19]. The study protocol adheres to the STROBE (Strengthening the Reporting of Observational Studies in Epidemiology) reporting guidelines and was formally registered (clinical study identifier: EudraCT number 2021-002611-54). The study was approved by the referring ethics committee (protocol number: 1407/CE Lazio1). Before taking part in the study, all subjects provided their informed consent. Participants who met the following inclusion criteria were eligible for the current analysis: histologically confirmed diagnosis of solid malignancy, locally advanced or metastatic extent of disease, at least one dose or course of an active antineoplastic treatment received before the third dose of tozinameran, no evidence of progressive disease on restaging performed within eight weeks before the third dose, and subsequent disease reassessment performed within six months of the third dose. The patients were monitored for the development of SARS-CoV-2 breakthrough infection at different time points (3, 6, and 12 months) or whenever it occurred first following the completion of the vaccination schedule. The coprimary endpoints of the study were the effects of IgG antibody titers against the receptor-binding domain of the SARS-CoV-2 spike protein (RBD-S1) on the development of breakthrough infections and the impact of COVID-19 outbreaks on cancer treatment failure from any cause. We reviewed all patient imaging to determine response rates as per the Response Evaluation Criteria in Solid Tumors (RECIST 1.1) [20]. The survival outcome was investigated in terms of vaccine-related time-to-treatment failure (V-TTF, which referred to the time elapsed from the date of booster dosing to permanent discontinuation of active treatment for any reason). Patients who had not withdrawn from cancer treatment undertaken before the third dose of tozinameran were censored at the time of the current analysis (cut-off date 7 March 2023).

### 2.2. Microbiological Assessments

Breakthrough infections were defined as laboratory-confirmed SARS-CoV-2 positivity by third-generation antigenic or polymerase chain reaction (PCR) tests. Commercially available diagnostic assays were used according to the standard public health protocols. All positive cases were reported to the government agency for epidemiological monitoring [21]. Although we did not provide any sequencing of SARS-CoV-2 strains, the predominant VOC was inferred from national epidemiological data on each outbreak case. The Delta (B.1.617.2) and Omicron (B.1.1.529) variants were considered in SARS-CoV-2 infections diagnosed until December 2021 and from January 2022 up to the date of the current analysis, respectively [22]. The levels of antibodies binding to RBD-S1 were determined four weeks after the third dose of tozinameran by the SARS-CoV-2 IgG II Quant assay on the ARCHITECT i2000sr automated platform (Abbott Laboratories, Diagnostics Division, Sligo, Ireland) according to the manufacturer’s instructions [23]. The results were provided as arbitrary units per milliliter (AU/mL) within a linear range expanded to 80,000 with an automated dilution. The serological titers obtained were converted from AU to binding antibody units (BAU) after WHO International Standards for anti-SARS-CoV-2 immunoglobulin testing were released (1 Abbott AU corresponds to 0.142 WHO BAU) [24].

### 2.3. Statistical Analysis

A mean with standard deviation was used to describe normally distributed variables, while a median with a 95% confidence interval (CI) or interquartile range (IQR) was reported for skewed variables. Comparative assessments were performed by applying Pearson’s χ2 test for categorical data and the Mann–Whitney U test or the Kruskal–Wallis test for continuous variables. A preliminary multivariate analysis was performed by adjusting a generalized linear model on the logarithmic (log) values of anti-RBD-S1 IgG titers as a function of predefined covariates, including sex, age, cancer type, Eastern Cooperative Oncology Group Performance Status (ECOG PS), treatment setting, corticosteroid therapy, type of active treatment, and COVID-19 infection. A receiver operating characteristic (ROC) curve was calculated to determine the sensitivity and specificity of anti-RBD-S1 titers in relation to the detection of SARS-CoV-2 breakthrough infections. The Youden index was applied to identify the optimal cut-point. A subsequent multivariate analysis was performed by adjusting a generalized linear model to estimate the odds ratio (OR) with a 95% CI of breakthrough infection as a function of predefined covariates, including sex, age, cancer type, Eastern Cooperative Oncology Group Performance Status (ECOG PS), treatment setting, corticosteroid therapy, type of active treatment, anti-RBD-S1 antibody levels, and additional booster dosing of vaccine after the third immunization. A Mantel–Cox log-rank test was used to compare the V-TTF outcomes of the different patient subgroups. Survival curves were visualized through the Kaplan–Meier method. A multivariate Cox regression model was applied to estimate the hazard ratio (HR) with a 95% CI of breakthrough infections in addition to the independent covariates described above. All of the tests performed were two-sided, and a *p*-value < 0.05 was considered significant. SPSS (IBM SPSS Statistics for Windows, version 23.0, Armonk, NY, USA) and Prism (GraphPad, version 9) software were used for the statistical evaluations and figure rendering, respectively.

## 3. Results

### 3.1. Patient Characteristics

The current analysis included 230 eligible patients who had received their third dose of tozinameran between 27 September and 7 October 2021. The majority of them were female (56.9%), with ECOG PS 0-1 (91.7%) and metastatic disease (97.8%). The most common types of solid malignancies were breast cancer (26.9%) and lung cancer (24.3%). Targeted therapy (39.6%) and cytotoxic chemotherapy (27.8%) were the most common treatments that were ongoing at the time of the third dose of tozinameran. Table 1 details the baseline characteristics of the enrolled patients.

### 3.2. Breakthrough Infections and Antibody Responses

**Figure 1:**
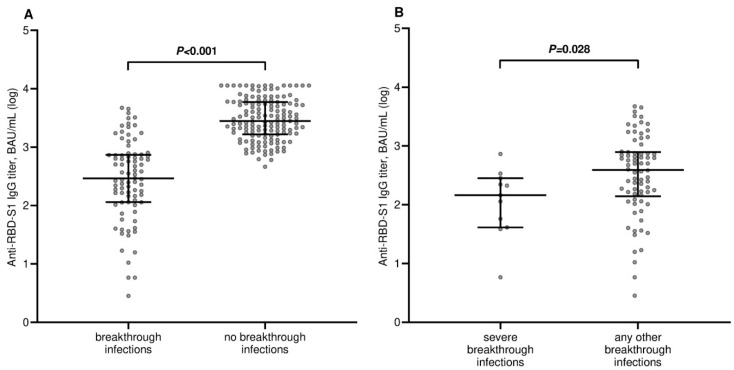
Comparison of scatter plot distributions and medians of antibody titers. (**A**) Comparison of antibody titers between breakthrough infection cases and non-cases. (**B**) Comparison of antibody titers between severe breakthrough infection cases and any other cases.RBD-S1, receptor-binding domain (RBD) of the SARS-CoV-2 Spike protein (S1); BAU, binding antibody unit; log, logarithmic values. Bars represent median values with a 95% confidence interval.

**Figure 2:**
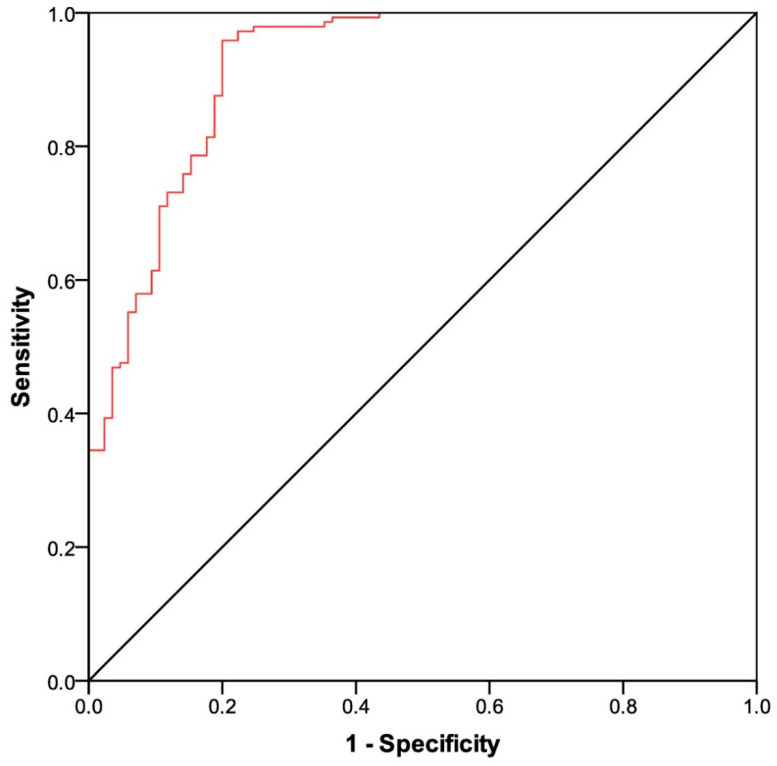
ROC curve analysis of anti-RBD-S1 IgG titers on the SARS-CoV-2 breakthrough infections AUC relative value: 0.92 (95% confidence interval 0.88–0.95), *p* < 0.001. ROC, receiver operating characteristic; RBD-S1, receptor-binding domain (RBD) of the SARS-CoV-2 Spike protein (S1); AUC, area under the curve.

At a median follow-up time of 16.3 months (IQR 5.4–17.1), 85 of the 230 (36.9%) patients developed a SARS-CoV-2 breakthrough infection with a median interval of 2.3 months (IQR 1.0–3.9) after the third immunization. According to the epidemiological curve of SARS-CoV-2 infections during the study period, the Delta and Omicron variants were likely causative in 52 (61.1%) and 33 (38.9%) of the outbreak cases, respectively. Most patients were asymptomatic or mildly symptomatic. Thirteen cases (15.3%) showed moderate symptoms that required home antiviral therapy. Eleven patients (12.9%) experienced severe symptoms that resulted in hospitalization. We observed only two deaths (2.3%) linked to COVID-19 outbreaks. In total, 9 of the 11 cases (81.8%) with a severe clinical course, and both COVID-19-related deaths occurred during the presumed wave of the Delta variant. The antibody titer after the third dose of tozinameran was significantly decreased in the breakthrough cases when compared to non-cases (291 BAU/mL (95% CI 210–505) vs. 2798 BAU/mL (95% CI 2323–3613), *p* < 0.001; Figure 1A). A similar estimate was observed in patients with severe infections (198 BAU/mL (95% CI 101–335) vs. 795 BAU/mL (95% CI 573–1040), *p* = 0.028; Figure 1B). As of March 2022, 47 patients (20.4%) received additional booster dosing of tozinameran and/or bivalent vaccination after the third immunization. In the multivariate analysis, only ECOG PS2 and COVID-19 infection were significantly correlated with an impaired antibody response (Supplementary Table S1). An ROC curve was calculated to determine the relationship between anti-RBD-S1 IgG titers after booster dosing and protection from SARS-CoV-2 outbreaks. The relative AUC value (0.92 (95% CI 0.88–0.95), *p* < 0.001) was considered valuable in predicting the likelihood of a negative outcome (Figure 2). The Youden index identified an optimal IgG titer cut-point of 803 BAU/mL, which was associated with a sensitivity of 0.95 and a specificity of 0.80 and dichotomization of recipients into low-responders (<803 BAU/mL) and high-responders (≥803 BAU/mL). In the multivariate analysis, a significant correlation was found between improved humoral responses and decreased odds of breakthrough infections. Moreover, it was observed that male sex, lung cancer diagnosis, cytotoxic chemotherapy, and their combination with biological agents were independent factors in raising the likelihood of outbreaks according to the same testing. Taking into account that 73 patients (31.7%) had already contracted SARS-CoV-2 breakthrough infections before any further booster dose of the vaccine became available, it is noteworthy that the receipt of this additional immunization was not correlated with a significant reduction in the odds of COVID-19 outbreaks (Table 2).

### 3.3. Time-to-Treatment Failure

At the time of the current analysis, we censored 82 patients (35.7%) who were still receiving ongoing anticancer treatment when the third dose of tozinameran was given. In the remaining 148 patients (64.7%), the reasons for discontinuing treatment were progression of the underlying disease (*n* = 129, 56.1%), direct consequences of COVID-19 infections (*n* = 8, 3.5%), refusal to continue (*n* = 5, 2.2%), death without evidence of disease progression which was regarded as a treatment failure event (*n* = 4, 1.7%), and other causes (*n* = 2, 0.8%). In a univariate subgroup analysis, patients who had developed an impaired humoral response following booster vaccination and reported SARS-CoV-2 breakthrough infections showed a significant decrease in V-TTF. At the same evaluation, patients with adverse prognostic features, including lung cancer diagnosis, ECOG PS2, and the need for corticosteroid treatment at immunosuppressive dosages, as well as those receiving cytotoxic chemotherapy, had worse outcomes (Supplementary Figure S1). In the multivariate analysis, all clinical variables except the diagnosis of lung malignancy retained their significant predictive values (Table 3).

## 4. Discussion

The current study provides a comprehensive characterization of SARS-CoV-2 infections in cancer patients on active treatment over an observation time frame of more than 12 months after the third vaccination. Additionally, we investigated their treatment outcomes in relation to COVID-19 outbreaks. To the best of our knowledge, this is the first research focusing on the disease outcomes of patients with solid malignancies undergoing boosters. The prospective design of our research, the substantial sample size, centralized serology testing, and the extended follow-up period make it possible to address multiple issues, including a precise depiction of SARS-CoV-2 breakthrough cases. In this regard, longitudinal clinical monitoring may result in a low likelihood bias of reporting only severe outbreak cases, as opposed to retrospective surveys.

The overall incidence of SARS-CoV-2 breakthrough infections in 37% of the observed cases is considerably higher in comparison to existing data of 13.6% within the same population [25]. Several underlying reasons may account for this disproportion. First, our research included periodic testing to identify even asymptomatic or mildly symptomatic cases, which constituted the majority of the patients (72%). If diagnostic tests had been performed only on those with more severe symptoms, these cases would probably not have been detected. Second, the prolonged detection period increased the incidence of cumulatively observed outbreak cases. In addition, we encountered the most intense waves of Delta (October 2021 to December 2021) and Omicron (January 2022 to July 2022) variants spread during the same time frame. It is worth noting that the prevalence of SARS-CoV-2 breakthrough infections in our series mirrors national epidemiological data [26]. Third, all participants in the study had advanced cancer disease and were receiving active antineoplastic treatment. Both conditions result in an underlying state of immunosuppression and may have rendered the patients more susceptible to outbreak infections [27]. The latter observation is consistent with the results of our multivariate analysis, which shows that treatments with stronger immunosuppressive potential, such as those involving cytotoxic chemotherapy, correlated independently with increased odds of infection. The same multivariable test also identified a poor humoral response as an independent predictor for COVID-19 outbreaks after the third dose of tozinameran. This is the first key finding of our study, which suggests that a weak antibody response exposes recipients to a higher infectious hazard despite booster vaccination. Although a significant number of participants experienced SARS-CoV-2 breakthrough infections, the course of infectious diseases was generally favorable. Fewer than 13% of the patients presented with severe symptoms requiring hospital admission, and there was an encouragingly less than 3% mortality rate due to COVID-19 sequelae. The finding that most breakthrough infections with a worse clinical course occurred during the putative Delta variant wave seems to confirm the lower virulence of the Omicron variant and the maintenance of booster vaccine protection against severe outcomes [28,29]. Although the limited number of severe infections precluded us from a multivariate analysis of clinical predictors, the significant benefit demonstrated in univariate comparison for those with enhanced antibody responses is in agreement with prior evidence [30].

The clinical effects of SARS-CoV-2 infections on the efficacy of various active treatments in solid tumors are currently unknown. The fact that most data are available in patients with hematologic malignancies makes it challenging to critically interpret our results [31,32]. According to the univariate analysis of this study, patients who developed a COVID-19 outbreak had a significantly shortened time-to-treatment failure after the third dose of tozinameran. Although less than 30% of outbreak cases required specific intervention, including antiviral therapy or hospitalization, and COVID-19 resulted in treatment discontinuation in only 4% of these patients, the multivariate analysis confirmed a negative predictive value of breakthrough infections. The impact on cancer treatment failure was independent and even stronger than adverse clinical features, such as poor ECOG PS and the need for prolonged corticosteroid therapy at immunosuppressive doses. Additionally, it is noteworthy that a weak antibody response was correlated with a worse outcome on the same multivariable testing. This is the second key finding of the current research, which suggests that patients with impaired humoral immunogenicity are not only at higher odds for SARS-CoV-2 breakthrough infection but also for early failure of active treatment.

The current study acknowledges several shortcomings, including but not limited to the following issues. The Vax-On-Third study, like all others of its kind, was designed to enroll large numbers of patients in a short time frame. While the need to address COVID-19-related emergencies may explain such an approach, this “all-comers” recruitment did not allow for adequate stratification of the participants, making the study prone to selection bias. We included a wide variety of malignancies, implying that clinical interactions between vaccination and several active treatments cannot be ruled out among patients with different types of cancer. The specific design of the present research also has an inherent immortal time bias, which results from the lapse between the initiation of active treatment and the third vaccination, potentially leading to an incorrect estimate of survival benefit [33]. In addition, we could not perform longitudinal monitoring of humoral responses to verify the effects of time-dependent waning immunity on breakthrough infections. Finally, our multivariate analysis included among the clinical covariates the receipt of additional boosters, such as the fourth dose of tozinameran [34] or bivalent vaccination [35], which were implemented as of March 2022. Since at that time 32% of the enrolled patients had already developed COVID-19 breakthrough infections, these pharmacological interventions cannot be considered applicable to the general study population. Evidence that additional booster doses may increase COVID-19 protection for cancer patients, even those who do not respond to the initial vaccine series, introduces potential confouders that we could not foresee at the beginning of this research [36].

## 5. Conclusions

Our findings strengthen the evidence that cancer patients with advanced disease undergoing active treatment maintain an increased risk of developing SARS-CoV-2 breakthrough infections despite third booster immunization. The protective efficacy of vaccination against severe infections is confirmed to be high, considering the low rate of COVID-19-related major sequelae and mortality, especially during the Omicron variant surge. Although most of them featured an indolent clinical course, COVID-19 outbreaks had a worsening effect on cancer therapy outcomes. Similar to hematologic malignancies, this result confirms that even mild SARS-CoV-2 infections can potentially disrupt anticancer treatment and thereby affect the survival of patients with advanced disease [37]. Monitoring humoral immunogenicity can help identify recipients at increased odds of breakthrough infections and those who should be prioritized for additional vaccinations to minimize the impact on their cancer treatment [38]. Our data suggest that antibody titers higher than 800 BAU/mL are viable correlates of immunologic protection from SARS-CoV-2 variant infections and severe symptomatic disease. The limitations of the current study emphasize that multivariable statistical comparisons may amplify false-positive results, the significance of which should therefore be considered exploratory and validated in independent cohorts.

## Figures and Tables

**Table 1:** Demographic and clinical characteristics of the study population.

**Characteristics**	**All Patients, *N* = 230 (100%)**
Age	
mean (SD), years	65.4 (10.5)
-≤55 years	46 (20.0%)
->55 years	184 (80.0%)
Sex	
-female	131 (56.9%)
-male	99 (43.1%)
ECOG PS	
0	98 (42.6%)
-1	113 (49.1%)
-2	19 (8.3%)
Cancer type	
-breast	62 (26.9%)
-lung	56 (24.3%)
-kidney	10 (4.3%)
-prostate	8 (3.5)
-colorectal	41 (17.8%)
-urothelial	8 (3.5%)
-pancreatic	10 (4.3%)
-gastric	5 (2.2%)
-skin (Melanoma or Merkelcell)	5 (2.2%)
-gynecological	9 (3.9%
-head and neck	2 (0.9%)
-brain	7 (3.0%)
-other ^a^	7 (3.0%)
Extent of disease	
-locally advanced	5 (2.2%)
-metastatic	225 (97.8%)
Treatment setting	
-advanced disease, first line	161 (70.0%)
-advanced disease, second or later line	69 (30.0%)
Type of last active treatment	
-targeted therapy	91 (39.6%)
-cytotoxic chemotherapy	64 (27.8%)
-immune checkpoint inhibitors	35 (15.2%)
-hormonal therapy	14 (6.1%)
-cytotoxic chemotherapy and biological therapy	26 (11.3%)
Time from last active treatment and booster vaccination	
-median (IQR), days	10 (2–18)
Corticosteroid therapy ^b^	41 (17.8%)
**Characteristics**	**All Patients, *N* = 230 (100%)**
Age	
mean (SD), years	65.4 (10.5)
≤55 years	46 (20.0%)
>55 years	184 (80.0%)
Sex	
female	131 (56.9%)
male	99 (43.1%)
ECOG PS	
0	98 (42.6%)
-1	113 (49.1%)
-2	19 (8.3%)
Cancer type	
breast	62 (26.9%)
lung	56 (24.3%)
kidney	10 (4.3%)
prostate	8 (3.5)
colorectal	41 (17.8%)
urothelial	8 (3.5%)
pancreatic	10 (4.3%)
gastric	5 (2.2%)
skin (Melanoma or Merkelcell)	5 (2.2%)
gynecological	9 (3.9%
head and neck	2 (0.9%)
brain	7 (3.0%)
other ^a^	7 (3.0%)
Extent of disease	
locally advanced	5 (2.2%)
metastatic	225 (97.8%)
Treatment setting	
advanced disease, first line	161 (70.0%)
advanced disease, second or later line	69 (30.0%)
Type of last active treatment	
targeted therapy	91 (39.6%)
cytotoxic chemotherapy	64 (27.8%)
immune checkpoint inhibitors	35 (15.2%)
hormonal therapy	14 (6.1%)
cytotoxic chemotherapy and biological therapy	26 (11.3%)
Time from last active treatment and booster vaccination	
median (IQR), days	10 (2–18)
Corticosteroid therapy ^b^	41 (17.8%)

SD, standard deviation; ECOG PS, Eastern Cooperative Oncology Group Performance Status; IQR, interquartile range. ^a^ Other cancer types included soft-tissue sarcoma, thymoma, testicular cancer, hepatobiliary cancer, esophageal cancer, and GIST; ^b^ corticosteroid therapy indicates ≥10 mg per day of prednisone or equivalent for at least 7 days at any time following the third vaccine dose.

**Table 2:** Multivariable analysis of breakthrough infection odds by the predefined clinical variables.

Covariates	OR (95% CI)	*p* Value
Sex		
-male vs. female	0.25 (0.07–0.85)	0.027
Age (years)		
->55 vs. ≤55	0.44 (0.13–1.44)	0.177
Cancer type		
-breast	1	-
-lung	14.62 (2.31–92.43)	0.004
-colorectal	4.84 (0.87–26.71)	0.07
-others	3.31 (0.66–16.50)	0.143
ECOG PS		
-0 ^a^	1	-
-1	1.35 (0.46–3.99)	0.581
-2	0.97 (0.13–7.32)	0.982
Treatment setting		
-advanced, first line vs. second or later line	2.22 (0.74–6.62)	0.343
Corticosteroid therapy		
-yes vs. no	1.50 (0.36–6.62)	0.572
Type of active treatment		
-targeted therapy ^a^	1	-
-cytotoxic chemotherapy	8.72 (2.14–35.48)	0.002
-immune checkpoint inhibitors	2.02 (0.40–10.23)	0.394
-hormonal therapy	1.37 (0.07–23.96)	0.827
-cytotoxic chemotherapy and biologics	13.47 (1.89–98.89)	0.009
Antibody response		
-high response vs. low response	0.004 (0.001–0.019)	<0.001
Additional booster vaccine dosing		
-yes vs. no	0.73 (0.21–2.23)	0.625

*p*-values derived from parametric 2-sided Wald’s χ2 test with Bonferroni (α = 0.01) correction for multiple comparisons. A two-sided *p*-value of <0.05 was considered statistically significant. ^a^ Reference category. OR, odds ratio; CI, confidence intervals; ECOG PS, Eastern Cooperative Oncology Group Performance Status. Corticosteroid therapy indicates ≥10 mg prednisone equivalent daily for at least 7 days at any time after the third dose of the vaccine. The high response indicates the subgroup of patients with an anti-RBD-S1 IgG titer ≥803 BAU/mL after the third dose of vaccine; the low response indicates the subgroup of patients with an anti-RBD-S1 IgG titer <803 BAU/mL after the third dose of the vaccine. Additional booster dosing of the vaccine included a fourth dose of tozinameran and/or bivalent vaccine.

**Table 3:** Analysis of time-to-treatment failure.

Covariates	Univariate Analysis		Multivariate Analysis	
	V-TTF, Months	*p* Value ^a^	HR (95% CI)	*p* Value ^b^
	(95% CI)			
Sex		0.832	-	-
-female	11.8 (7.1–16.6)			
-male	7.6 (4.3–10.9)			
Age (years)		0.477	-	-
-≤55	5.4 (2.1–8.7)			
->55	9.8 (6.0–13.5)			
Cancer type		0.001		0.115
-others	12.1 (8.2–16.0)		1	
-lung cancer	5.0 (3.1–7.0)		1.36 (0.92–2.00)	
ECOG PS		<0.001		<0.001
-0 or 1	11.8 (7.8–15.8)		1	
-2	2.4 (1.6–3.1)		3.00 (1.75–5.14)	
Treatment setting		0.171	-	-
-advanced, first line	11.8 (7.4–16.21)			
-advanced second or later line	6.4 (4.2–8.6)			
Corticosteroid therapy		<0.001		0.014
-no	12.2 (7.7–16.6)		1	
-yes	3.0 (1.9–4.0)		1.68 (1.11–2.55)	
Type of active treatment		<0.001		0.039
-cytotoxic-chemotherapy-based	5.2 (3.8–6.6)		1	
-any other	14.4 (10.4–16.3)		1.44 (1.01– 2.06)	
COVID-19 infection		<0.001		<0.001
-no	16.2 (14.3–17.0)		1	
-yes	3.1 (2.3–3.6)		5.66 (3.29–9.74)	
Antibody response		<0.001		0.017
-high response	14.6 (11.9–16.3)		1	
-low response	3.6 (3.0–4.5)		1.88 (1.11–3.19)	

^a^ *p*-values derived from the Mantel–Cox log-rank test to compare the outcomes of different patient subgroups; ^b^ *p*-values derived from the multivariate Cox regression model with the parametric 2-sided Wald’s χ2 test and Bonferroni (α = 0.01) correction for multiple comparisons. A two-sided *p*-value of <0.05 was considered statistically significant. V-TTF, vaccine-related time-to-treatment failure; CI, confidence interval; HR, hazard ratio; ECOG PS, Eastern Cooperative Oncology Group Performance Status. Corticosteroid therapy indicates ≥10 mg prednisone equivalent daily for at least 7 days at any time after the third dose of the vaccine. The high response indicates the subgroup of patients with an anti-RBD-S1 IgG titer ≥803 BAU/mL after the third dose of the vaccine; the low response indicates the subgroup of patients with an anti-RBD-S1 IgG titer <803 BAU/mL after the third dose of vaccine.

## Data Availability

The datasets generated and analyzed during the current study are available from the corresponding author upon reasonable request.

## Supplementary Materials

The following supporting information can be downloaded at: https://www.mdpi.com/article/10.3390/curroncol30050386/s1, Figure S1: Vaccine-related time-to-treatment failure depending on clinical variables; Table S1: Analysis of antibody response by predefined clinical variables.
