# Supplementary material for: Effects of Antibody Response after Booster Vaccination on SARS-CoV-2 Breakthrough Infections and Disease Outcomes in Advanced Cancer Patients: A Prospective Analysis of the Vax-on-Third Study
Source: Curr Oncol. 2023 May 17;30(5):5103–15. doi: 10.3390/curroncol30050386 (PMC10217296; doi:10.3390/curroncol30050386)
Supplement: Supplementary file 1 [file curroncol-30-00386-s001.zip › curroncol-2362371-supplementary.pdf]

SUPPLEMENTARY MATERIAL

Effects of antibody response after booster vaccination on SARS-CoV-2 breakthrough infections and disease outcomes in advanced cancer patients: A prospective analysis of the Vax-On-Third study.

Fabrizio Nelli [1,*], Agnese Fabbri [1], Antonella Virtuoso [1], Diana Giannarelli [2], Julio Rodrigo Giron Berrios [1], Eleonora Marrucci [1], Cristina Fiore [1], Marta Schirripa [1], Carlo Signorelli [1], Mario Giovanni Chilelli [1], Francesca Primi [1], Gloria Pessina [3], Federica Natoni [3], Maria Assunta Silvestri [4], and Enzo Maria Ruggeri [1]

[1] Department of Oncology and Hematology, Medical Oncology Unit, Central Hospital of Belcolle, Viterbo, Italy

[2] Biostatistics Unit, Scientific Directorate, Fondazione Policlinico Universitario A. Gemelli, IRCCS, Rome, Italy

[3] Department of Oncology and Hematology, Molecular Biology and Covid Diagnostics, Central Hospital of Belcolle, Viterbo, Italy

[4] Department of Oncology and Hematology, Microbiology and Virology Unit, Central Hospital of Belcolle, Viterbo, Italy

* **Correspondence:** Fabrizio Nelli, MD; Department of Oncology and Hematology, Medical Oncology Unit, Central Hospital of Belcolle, Strada Sammartinese snc, 01100 Viterbo, Italy, Phone +390761339055, Fax +390761339039, e-mail: fabrizio.nelli@asl.vt.it, ORCID iD: 0000-0001-8374-1362

Supplementary Table S1. Analysis of antibody response by predefined clinical variables

	Univariate analysis		Multivariate analysis	
Covariates	Anti-RBD-S1 IgG titer (BAU), median of absolute values (95% CI)	P value[†]	Beta (95% CI), anti-RBD-S1 IgG titer logarithmic values	P value[‡]
Sex		0.002		
- female	2121 (1698-2762)		1.00	-
- male	1158 (747-1937)		-0.12 (-0.27 to 0.02)	0.105
Age (years)		0.237		
- ≤55	1295 (755-2043)		1.00	-
- >55	1862 (1385-2323)		0.01 (-0.15 to 0.16)	0.933
Cancer type		<0.001		
- breast[a]	3226 (2323-4134)		1.00	-
- lung	767 (505-1252)		-0.09 (-0.31 to 0.13)	0.421
- colorectal	2064 (1256-3130)		0.15 (-0.07 to 0.38)	0.188
- others	1343 (926-2334)		-0.13 (-0.32 to 0.06)	0.194
ECOG PS		0.001		
- 0[a]	2353 (1768-3531)		1.00	-
- 1	1578 (1188-1921)		0.03 (-0.10 to 0.17)	0.637
- 2	282 (101-2307)		-0.27 (-0.53 to -0.01)	0.036
Treatment setting		0.978		
- advanced, first line	1746 (1213-2182)		1.00	-

- second or later line	2067 (1331-2531)		-0.10 (-0.24 to 0.04)	0.177
Corticosteroid therapy		0.001		
- no	1981 (1569-2323)		1.00	-
- yes	767 (291-1637)		-0.12 (-0.30 to 0.05)	0.170
Type of active treatment		0.012		
- targeted therapy[a]	2019 (1358-3108)		1.00	-
- cytotoxic chemotherapy	1289 (737-2327)		0.14 (-0.01 to 0.31)	0.082
- immune checkpoint inhibitors	1412 (1158-1981)		0.14 (-0.07 to 0.35)	0.208
- hormonal therapy	4122 (2934-5836)		0.12 (-0.15 to 0.40)	0.385
- cytotoxic chemotherapy and biologics	1209 (390-1843)		-0.04 (-0.28 to 0.20)	0.737
COVID-19 infection		<0.001		
- no	2798 (2323-3613)		1.00	-
- yes	291 (210-505)		-0.96 (-1.10 to -0.81)	<0.001

[†]P values derived from Mann-Whitney *U* test or Kruskal-Wallis test; [‡]P values derived from parametric 2-sided Wald's $\chi 2$ test with Bonferroni (α =0.01) correction for multiple comparisons. A two-sided P value of <0.05 was considered statistically significant.

[a] Reference category. RBD-S1, receptor binding domain of SARS-CoV-2 spike protein; BAU, binding antibody units; OR, odds ratio; CI, confidence intervals; ECOG PS, Eastern Cooperative Oncology Group Performance Status. Corticosteroid therapy indicates ≥10 mg prednisone equivalent daily for at least 7 days at any time after the third dose of vaccine. High response indicates the subgroup of patients with an anti-RBD-S1 IgG titer ≥803 BAU/mL after the third dose of vaccine; low response indicates the subgroup of patients with an anti-RBD-S1 IgG titer <803 BAU/mL after the third dose of vaccine.

Supplementary Figure S1. Vaccine-related time-to-treatment failure depending on clinical variables

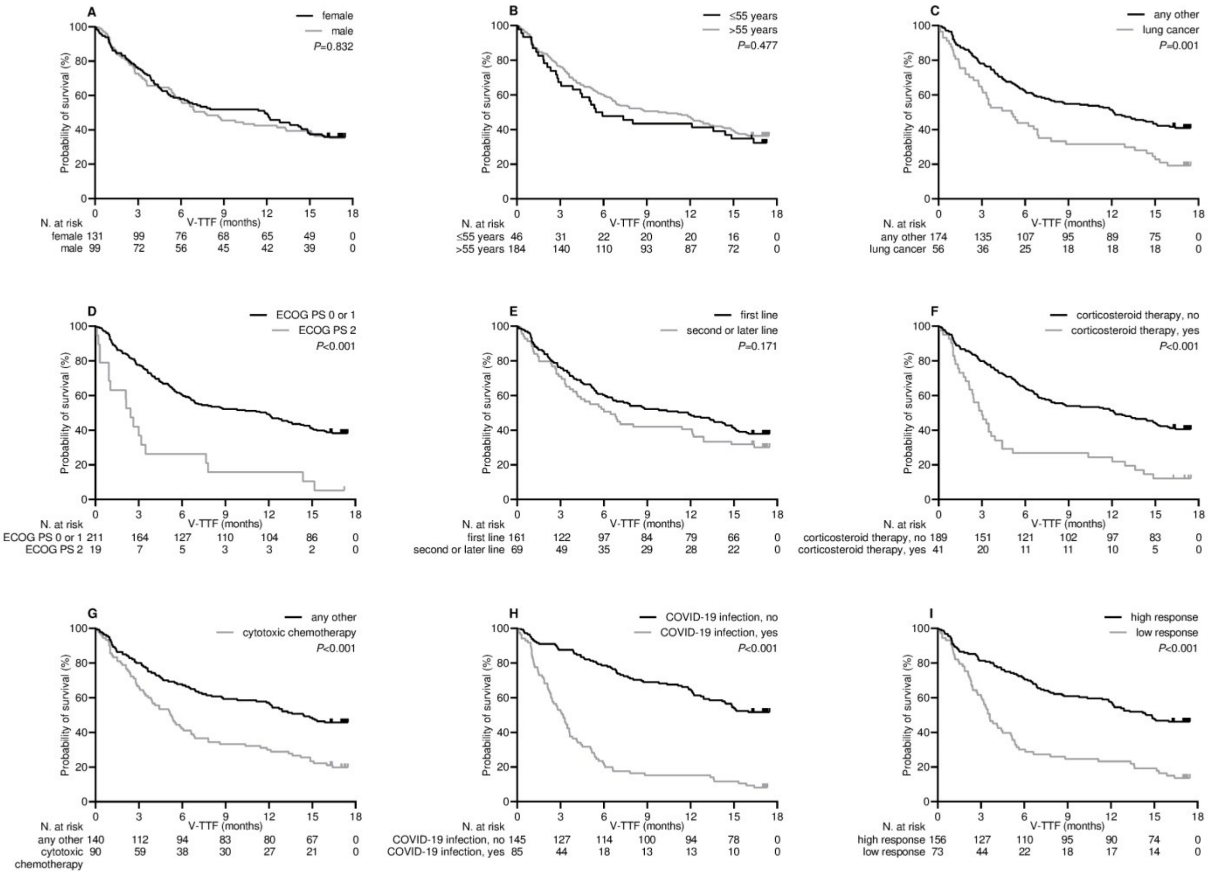

A. Sex: female vs male; B. Age group: ≤55 years vs >55 years; C. any other cancer diagnosis vs lung cancer diagnosis; D. Eastern Cooperative Oncology Group Performance Status (ECOG PS): 0 or 1 vs 2; E. Treatment setting: advanced, first line vs advanced, second or later line; F. Corticosteroid therapy (≥10 mg prednisone equivalent daily for at least 7 days at any time after the third dose of vaccine): no vs yes; G. Type of active treatment: any other vs cytotoxic chemotherapy based; H. COVID-19 infection: no vs yes; I. Level of antibody response: high response (subgroup of patients with an anti-RBD-S1 IgG titer ≥803 BAU/mL after the third dose of vaccine vs low response (subgroup of patients with an anti-RBD-S1 IgG titer <803 AU/mL after the third dose of vaccine.
